# Genetic Mapping and Exome Sequencing Identify Variants Associated with Five Novel Diseases

**DOI:** 10.1371/journal.pone.0028936

**Published:** 2012-01-17

**Authors:** Erik G. Puffenberger, Robert N. Jinks, Carrie Sougnez, Kristian Cibulskis, Rebecca A. Willert, Nathan P. Achilly, Ryan P. Cassidy, Christopher J. Fiorentini, Kory F. Heiken, Johnny J. Lawrence, Molly H. Mahoney, Christopher J. Miller, Devika T. Nair, Kristin A. Politi, Kimberly N. Worcester, Roni A. Setton, Rosa DiPiazza, Eric A. Sherman, James T. Eastman, Christopher Francklyn, Susan Robey-Bond, Nicholas L. Rider, Stacey Gabriel, D. Holmes Morton, Kevin A. Strauss

**Affiliations:** 1 Clinic for Special Children, Strasburg, Pennsylvania, United States of America; 2 Department of Biology and Biological Foundations of Behavior Program, Franklin & Marshall College, Lancaster, Pennsylvania, United States of America; 3 The Broad Institute, Boston, Massachusetts, United States of America; 4 Department of Biology, Swarthmore College, Swarthmore, Pennsylvania, United States of America; 5 Department of Pathology and Laboratory Medicine, School of Medicine and Public Health, University of Wisconsin, Madison, Wisconsin, United States of America; 6 College of Medicine, University of Vermont, Burlington, Vermont, United States of America; 7 Lancaster General Hospital, Lancaster, Pennsylvania, United States of America; Innsbruck Medical University, Austria

## Abstract

The Clinic for Special Children (CSC) has integrated biochemical and molecular methods into a rural pediatric practice serving Old Order Amish and Mennonite (Plain) children. Among the Plain people, we have used single nucleotide polymorphism (SNP) microarrays to genetically map recessive disorders to large autozygous haplotype blocks (mean = 4.4 Mb) that contain many genes (mean = 79). For some, uninformative mapping or large gene lists preclude disease-gene identification by Sanger sequencing. Seven such conditions were selected for exome sequencing at the Broad Institute; all had been previously mapped at the CSC using low density SNP microarrays coupled with autozygosity and linkage analyses. Using between 1 and 5 patient samples per disorder, we identified sequence variants in the known disease-causing genes *SLC6A3* and *FLVCR1*, and present evidence to strongly support the pathogenicity of variants identified in *TUBGCP6*, *BRAT1*, *SNIP1*, *CRADD*, and *HARS*. Our results reveal the power of coupling new genotyping technologies to population-specific genetic knowledge and robust clinical data.

## Introduction

The Plain populations of Pennsylvania are descended from small groups of Swiss immigrants who organized into multiple endogamous demes that have remained genetically isolated over the last 12–14 generations [1], [2]. Certain recessive disorders are highly concentrated in Plain sects [3], [4]. The overwhelming majority (>99%) of affected individuals are homozygous for their respective pathogenic variant, which resides within a relatively large, homozygous haplotype block. We have exploited this knowledge to map dozens of recessive conditions using low-density (i.e. 10,000 and 50,000 marker) single nucleotide polymorphism (SNP) microarrays with as few as two patients [5]. This is an efficient, low-cost strategy [2].

The ease of genetic mapping is counterbalanced by the difficulty of disease gene identification. Shared homozygous blocks among affected individuals tend to be large (mean 4.4 Mb) and contain dozens or hundreds of genes [5], [6], [7], [8], [9], [10], [11]. Large gene lists are significant obstacles, particularly if expression and functional data provide few clues to prioritize the list. Since 2004, we have mapped loci for 28 genetic disorders within Amish and Mennonite demes. For 11 (40%) of these, we could not identify the causative gene as no pathogenic variants were found after sequencing all high-priority candidate genes within the mapped interval.

Exome sequencing has recently been shown to expedite disease gene discovery [12], [13], [14], [15], [16], [17], [18], [19], [20], [21], [22], [23], [24], [25], [26], [27], [28]. In a pilot study to investigate the utility of exome sequencing, the Clinic for Special Children and the Broad Institute initiated a collaboration to combine thorough phenotyping, autozygosity mapping, and exome sequencing. Within 12 months, we identified pathogenic variants for seven disorders, six of them novel. To delineate the functional consequences of these variants, we designed and executed studies of mutant protein expression and function. This work highlights the extraordinary potential of next generation technologies for the investigation of monogenic disease among appropriately selected individuals, families, and communities. It provides a realistic model for using next generation sequencing strategies in everyday clinical practice.

## Results

### Phenotypes

The phenotypes are summarized in Table 1. Detailed descriptions of each disorder are presented below.

**Table 1:** Phenotype Summary.

Disease	Group	OMIM	Clinical Synopsis
Infantile parkinsonism-dystonia	M	–	Infantile-onset rigidity, dystonia, or chorea
			Dopamine non-responsive Parkinsonism
			Progressive frontal lobe degeneration
Lethal neonatal rigidity and seizure syndrome	A	–	In utero myoclonic spasms
			Neonatal-onset intractable focal seizures
			Congenital rigidity
			Dysautonomia (hypothermia, apnea, bradycardia, SIDS)
Mental retardation, non-syndromic	M	–	Delayed language development
			Mild-moderate mental retardation
Microcephaly with chorioretinopathy	M	251270	Congenital pachygyric microcephaly
			Global developmental delay
			Chorioretinopathy and retinal detachment
Posterior column ataxia with retinitis pigmentosa	M	609033	Impaired propioception
			Retinitis pigmentosa
Symptomatic epilepsy and skull dysplasia	A	–	Global developmental delay
			Intractable epilepsy
			Skull dysplasia
Usher syndrome	A	–	Retinitis pigmentosa
			Progressive sensorineural hearing loss
			Episodic psychosis

#### Infantile Parkinsonism-dystonia syndrome

The phenotype and gene defect for infantile parkinsonism-dystonia have been described elsewhere [29]. Briefly, our patient developed irritability and feeding difficulties soon after birth. Generalized rigidity and dystonia developed during early infancy, impeding motor development, and evolving into severe rigid Parkinsonism by late childhood. The proband cannot speak or use her hands to communicate, and it has thus been difficult to assess cognitive function or thought content. Brain structure is normal. In cerebrospinal fluid, homovanillic acid (HVA) is elevated, 5-hydroxyindoleacetic acid (5HIAA) is normal, and the concentration ratio of HVA to 5HIAA is 6.8–13.2 mol:mol (normal 1 – 3.7 mol:mol). Treatment with haloperidol, tetrabenazine, levodopa-carbidopa, trihexyphenidyl, and tyrosine restriction have been ineffective.

#### Lethal neonatal rigidity and multifocal seizure syndrome

Episodic jerking begins *in utero*. Newborns have small heads (1.5 to 2 SD below normal for age), overlapping cranial sutures, small or absent fontanelles, and depressed frontal bones. Hands are fisted and extreme axial and limb rigidity prohibit volitional movements and tendon reflexes. Brief focal jerks of the tongue, face and arms are prominent soon after birth and occur in a nearly continuous sequence throughout each child's short life. Neuroimaging is normal or reveals mild hypoplasia of the frontal lobes. Electroencephalograms show bilateral medium-high voltage spikes over temporal and central regions, frequent multifocal seizures, background slowing, and no posterior rhythm. Seizures are only partially responsive to anticonvulsants and not affected by high-dose pyridoxine.

**Figure 1:**
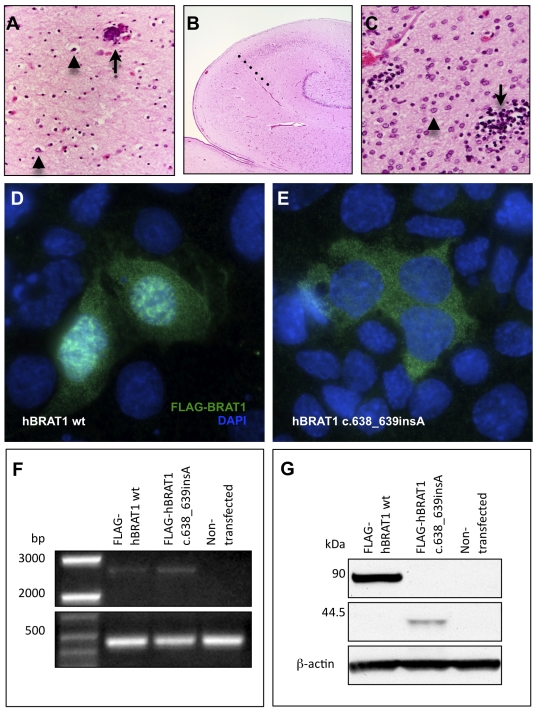
Corticobasal degeneration in the brain of an infant who died from a homozygous *BRAT1* mutation. (**A**) Throughout frontal, occipital and temporal cortex, there is marked neuronal loss, gliosis with astrocytes (arrowheads) and swollen oligodendroglia. The arrow indicates a perivascular microcalcification (superior frontal gyrus, deep cortex, 10×). (**B**) The anterior hippocampus is smaller than expected and there is neuronal loss and gliosis in zone CA-1 (Sommer's sector), demarcated from the CA-2 sector by the dotted line (4×). (**C**) At 60× magnification, the putamen shows a paucity of neurons, abundant Alzheimer Type 2 astrocytes (arrowhead) and scattered microglial nodules (arrow). Heterologous overexpression of N-terminal FLAG-tagged human BRAT1 (**D**) and hBRAT1 c.638_639insA (**E**) in mouse IMCD3 cells. Wild-type Brat1 localizes to the nucleus and cytoplasm of mIMCD3 cells. Mutant Brat1 (c.638_639insA) does not localize to the nucleus and instead forms punctate aggregations in the cytoplasm. Similar results were obtained in hARPE-19 cells (data not shown). (**F**) RT-PCR demonstrating the stability of overexpressed human BRAT1 transcripts (∼2.6 kb) in hARPE-19 cells. A B-actin amplicon (∼450 bp) was used as a loading control on the same gel. (**G**) Western blot of lysates from human ARPE-19 cells transiently transfected with wt hBRAT1 displaying FLAG-hBRAT1 fusion protein at ∼90 kDa or with hBRAT1 c.638_639insA displaying the truncated FLAG-hBRAT1 mutant fusion protein at ∼44.5 kDa (FLAG-tag and linker = 3.1 kDa). B-actin was labeled as a loading control.

Affected children have stagnant head growth, remain visually inattentive, do not feed independently, and make no developmental progress. They have frequent spontaneous apnea and bradycardia that uniformly culminates in cardiopulmonary arrest before age 4 months. The brain of a child who died at 4 weeks of age weighed 382 grams (expected for age 433 +/− 50 g) but was otherwise normally developed. Primary lesions were localized to most regions of the corpus striatum and cerebral cortex with relative sparing of the anterior caudates and parietal lobes. Lesions consisted of neuronal loss associated with a striking microglial reaction and proliferation of Alzheimer type 2 astrocytes (Figure 1, A–C).

#### Microcephaly with chorioretinopathy

**Figure 2:**
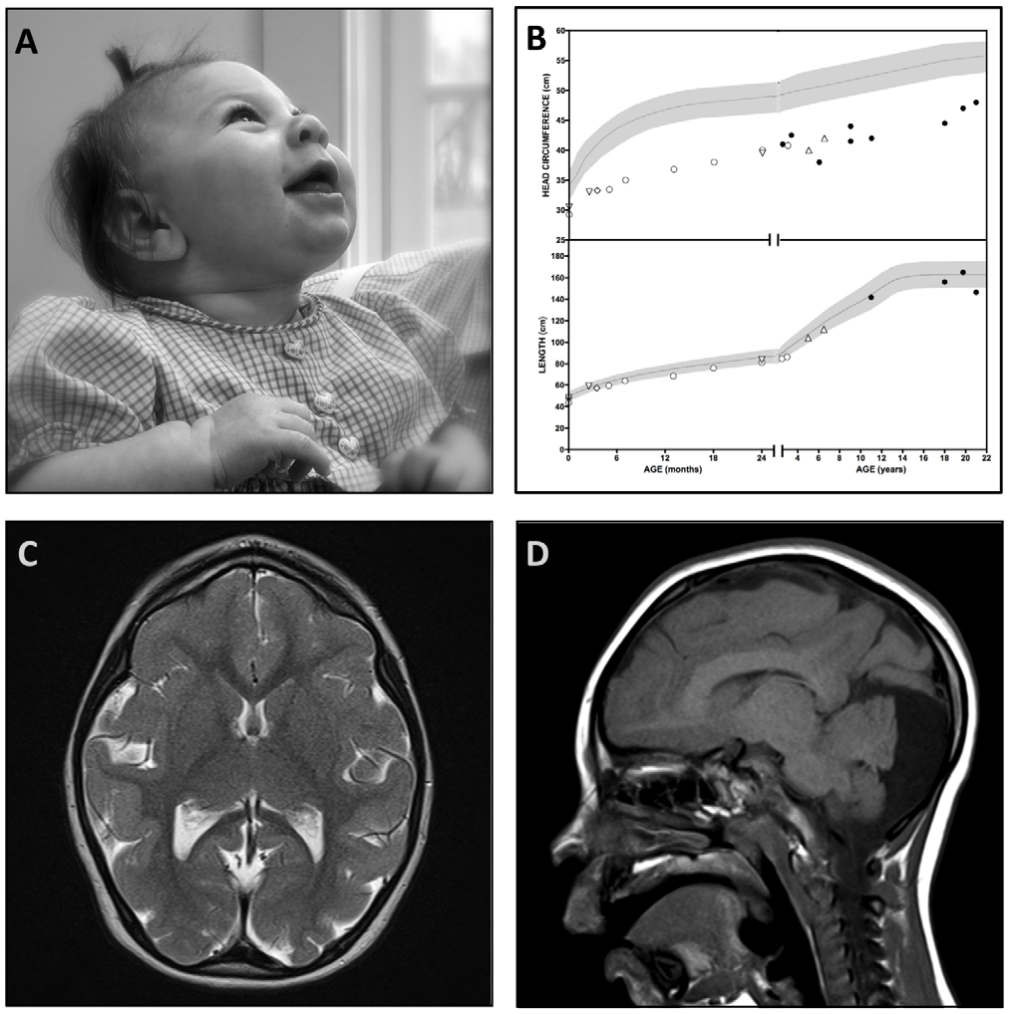
Microcephaly and chorioretinopathy due to a homozygous *TUBGCP6* mutation. (**A**) An affected infant has marked microcephaly (>4SD below normal), a receding forehead, diminutive anterior fontanelle, and sutural ridging. She has cognitive delay and visual impairment but is socially engaged. (**B**) Head circumference and length plots for Mennonite microcephaly patients. (**C**) Brain magnetic resonance imaging (MRI) shows diffuse pachygyria, normal myelination, and (**D**) a hypoplastic cerebellar vermis.

The phenotype was originally described by Victor McKusick [30]. All patients are born with microcephaly, a sloping forehead, diminutive anterior fontanelle, and sutural ridging (Figure 2, A). Head circumference is more than 4 standard deviations below normal at birth and remains so into adulthood (Figure 2, B). Affected neonates transition well and feed normally. Children walk independently between 14 and 36 months of age and language emerges at an appropriate age but remains rudimentary. Cognitive impairment ranges from moderate (adult mental age 8–10 years) to severe (adult mental age <6 months); intelligence quotients of two young patients were 60 and 62 (normal 100±15). Two of nine patients (22%) have epilepsy: one started having drop attacks in late childhood and another developed nocturnal epilepsy as an adult.

Magnetic resonance imaging shows diffuse pachygyria (Figure 2, C). The cerebral hemispheres are small relative to the cerebellum, which has a hypoplastic vermis (Figure 2, D). Although myelin volume appears reduced, it has normal signal quality. The surface area of the corpus callosum is approximately half that of an age-matched control child (2.75 cm^2^ versus 5.61 cm^2^).

Visual impairment becomes evident during the first year of life. The retina and choroid are underdeveloped and have focal defects that reveal bare sclera. Just posterior to the equator of the eye, much of the retina has a scalloped appearance that suggests focal areas of arrested development. The more anterior parts of the retina, near the periphery and pars plana, have a grayish hue and diminutive vasculature similar to retinopathy of prematurity. Condensations of vitreous may attach to the retina in transition regions between scalloped and gray tissue, marking points of traction for retinal detachment.

#### Non-syndromic mental retardation

The phenotype is typical of that described for other forms of non-syndromic mental retardation. All milestones are mildly delayed and cognitive function remains significantly impaired, precluding independent living and self-care. Speech is rudimentary but articulate. Affected individuals are not autistic.

#### Posterior column ataxia and retinitis pigmentosa

The AXPC1 phenotype has been described elsewhere [31]. Tunnel-like visual loss and photophobia begin early in childhood when fundoscopy reveals signs of non-spiculated retinitis pigmentosa and cellophane maculopathy. As vision deteriorates throughout adolescence, patients might develop posterior subcapsular cataracts. Motor milestones are slightly delayed (independent ambulation by 18 months).

Sensory ataxia, wide-based gait, and Rombergism emerge by age 4. Signs of sensory neuropathy include pan-areflexia, stocking-glove loss of vibration and position sense, astereognosia, agraphesthesia, and blunted sensation of applied force (e.g. accidentally crushing paper cups). Muscle tone, power, and electromyography are normal. MRI reveals T2 signal hyperintensity running the length of the dorsal spinal cord. Sensory sural nerve action potentials and H waves are absent. Some patients develop focal epilepsy marked by interictal focal spike-wave discharges. Cognitive function is normal.

#### Symptomatic epilepsy and skull dysplasia

**Figure 3:**
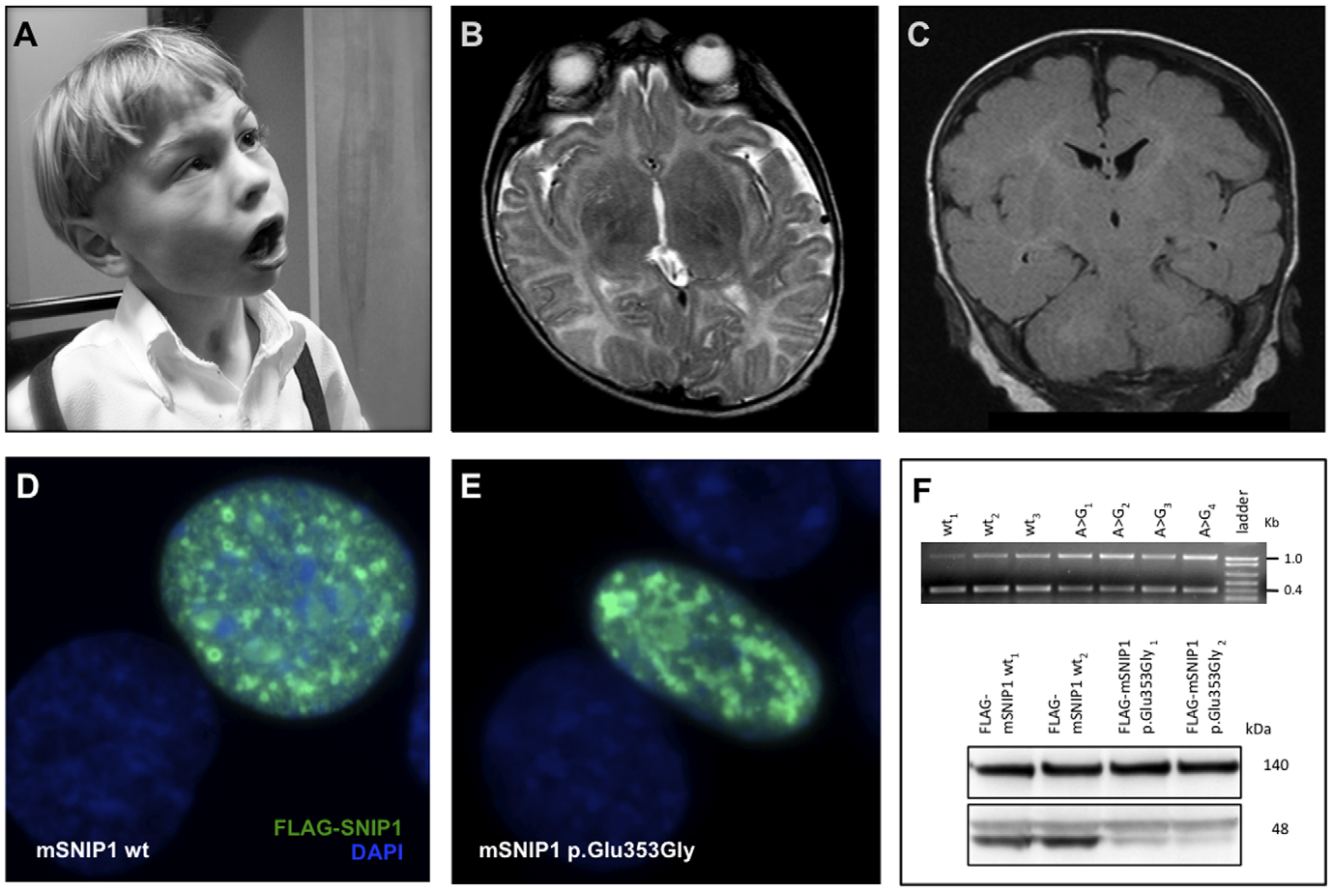
Symptomatic epilepsy and skull dysplasia due to a homozygous *SNIP1* mutation. (**A**) Two affected brothers presented with severe psychomotor delay, intractable seizures, bulbous nose, wide mouth and tongue, broad jaw with protuberant angles, short hands, short tapered fingers, and broad thumbs. (**B,C**) Brain MRI (B, axial T2; C, coronal T1) MRI showed enlarged ventricles, a thin corpus callosum, hypomyelination, and an irregular, undulating skull surface. (**D**) Mouse FLAG-SNIP1 (wt) fusion protein, when transiently overexpressed in mIMCD3 cells, localizes to the nucleus in a punctate pattern consistent with transcriptional complexes. (**E**) Mouse FLAG-SNIP1 (p.Glu353Gly) localizes to the nucleus, but with a more aggregated distribution. (**F**) *Top* – Reverse-transcriptase PCR from three wild-type mSNIP1-transfected samples and four c.1058A>G mSNIP1-transfected samples. mSNIP1 amplicon – 400 bp. mGAPDH (loading control) – 1037 bp amplicon. *Bottom* – Western blot of lysates from mIMCD3 cells transiently transfected with wt (lanes wt_1&2_) or c.1058A>G (lanes p.Glu353Gly_1&2_) mSNIP1 displaying the FLAG-mSNIP fusion protein at ∼48 kDa. The 140-kDa non-specific band was used as a loading control. Data shown are two out of four replicate sets of transfections.

Affected neonates are hypotonic and feed poorly. Dysmorphic features that evolve over time include a bulbous nose, wide mouth and tongue, broad jaw with protuberant angles, short hands, short tapered fingers, and broad thumbs (Figure 3, A). Affected children have severe psychomotor delay and do not learn to walk or speak. Many retain use of their hands to scoot, maneuver a wheelchair, or gesture. Some children show behavioral responses to language, but they do not socially engage or follow verbal commands.

Examination typically reveals a subdued child with strabismus, slow horizontal nystagmus, hypotonia, and weak or absent tendon reflexes. Magnetic resonance imaging reveals ventriculomegaly, thin corpus callosum, white matter abnormalities, and an undulating or “lumpy” skull surface (Figure 3, E). The cortical ribbon follows the irregular skull contour (Figure 3, E–F). Multifocal spike-wave discharges from central, occipital, and temporal regions typically begin by 6 months of age and are accompanied by focal or generalized seizures that can manifest as dystonic posturing, drop attacks, myoclonic jerks, or generalized tonic-clonic events. Multiple intractable seizure types can afflict an individual patient. Physical anomalies found in some patients include subglottic stenosis, aortic stenosis, bicuspid aortic valve, umbilical hernia, and hydrocele.

#### Usher syndrome

Growth and development are normal during infancy. Visual impairment becomes evident during early childhood with the emergence of fine horizontal nystagmus, light aversion, and optic pallor. As vision deteriorates, fundoscopic exam reveals marked attenuation of retinal arteries and veins, pigmentary changes and a cellophane-like reflex that produce “bull's eye” maculae and diffuse pigmentary stippling of the peripheral retinae, consistent with retinitis pigmentosa. This constellation suggests a combination of optic nerve disease, retinal dystrophy, and cone dysfunction. Patients are typically blind by the second or third decade of life but the pace of visual deterioration is highly variable.

We do not have auditory data from affected newborns, but some auditory function is present during infancy and deteriorates during early childhood; all five evoked auditory waveforms are absent by age 5. Amplifiers or cochlear implants can partially restore hearing. Patients have delayed gross motor development, hyperactive patellar tendon reflexes, mild truncal ataxia, and a wide-based gait. In contrast, upper limb coordination (allowing for visual impairment) and reflexes, peripheral nerve function, strength, tone, and intelligence are normal. Based on the current classification scheme, this condition is most consistent with the type III variant of Usher syndrome, which is characterized by progressive vision and hearing loss during early childhood years.

Infectious illnesses may provoke vivid visual hallucinations (the Charles Bonnet syndrome). These attacks begin during early childhood and may be accompanied by nonsensical speech, inappropriate laughter, repetitive eye blinking, or psychomotor agitation. In one case, acute psychosis merged into a deep catatonia that lasted several days. Hallucinations typically respond to anti-psychotic medications (e.g. haloperidol, thorazine) and are sometimes associated with transient myopathy (elevated serum creatine kinase). Rarely, children die suddenly and unexpectedly during an illness. These are presumably cardiac events, but routine electrocardiogram and 24-hour Holter monitor results have been normal.

### Genetic Mapping

**Figure 4:**
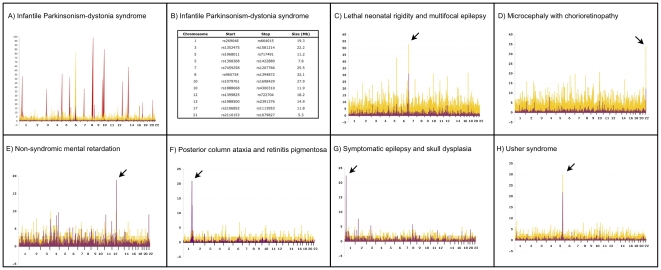
Genetic mapping of seven Plain disorders. The results of autozygosity mapping using Affymetrix GeneChip 10 K or 50 K SNP microarrays are plotted for each disorder. The x-axis depicts chromosomal location on autosomes. Yellow peaks represent the number of contiguous homozygous SNPs shared by affected individuals and the purple peaks depict location scores. (**A**) Autozygosity mapping of two affected individuals identified a single, large block of homozygosity on chromosome 6 (yellow peak). Genotyping of 6 unaffected siblings excluded this homozygous block, but identified 12 genomic regions greater than 5 Mb in size (red peaks) that were consistent with linkage in the family. (**B**) List of genomic regions consistent with linkage in the single nuclear family with infantile parkinsonism-dystonia syndrome. Panels **C–H** provide mapping plots for the other 6 disorders. For two disorders (**C,D**), 50 K microarrays were used after 10 K microarrays failed to unequivocally localize the disease gene. The other four disorders (**E–H**) were mapped with 10 K microarrays.

We chose to study 15 patients representing the seven discrete phenotypes described above (Table 1). All patients were originally genotyped using 10,000 (10 K) and/or 50,000 (50 K) single nucleotide polymorphism (SNP) microarrays as previously described [5], [6], [7], [8], [9], [10], [11]. Using between 2 and 9 samples, we genetically mapped five disorders using 10 K SNP arrays; four of these were localized with ease using *multiple affected children from separate sibships* (Figure 4, panels E–H). For two conditions, a shared homozygous block could only be identified at 50 K SNP resolution (see below). Shared autozygous blocks were an average of 4.4 Mb (range 1.6–8.4 Mb) and contained a mean of 79 (range 22–187) genes. The genetic mapping studies are summarized in Table 2.

**Table 2:** Genetic Mapping Summary.

Disease	Sample Size	Chr	SNP Start	SNP Stop	Size (Mb)	Genes
**Infantile parkinsonism-dystonia**	2	Many	–	–	–	–
**Lethal neonatal rigidity and seizure syndrome**	3	7	rs2251235	rs765728	1.6	31
**Mental retardation, non-syndromic**	6	12	rs709228	rs1369822	3.6	46
**Microcephaly with chorioretinopathy**	6	22	rs2157310	rs7364173	1.8	22
**Posterior column ataxia with retinitis pigmentosa**	9	1	rs10494916	rs9308430	4.2	59
**Symptomatic epilepsy and skull dysplasia**	6	1	rs2153337	rs2225251	6.6	126
**Usher syndrome**	3	5	rs2603014	rs325229	8.4	187

For *infantile Parkinsonism-dystonia syndrome*, the proband was one of the first patients to be examined at the CSC nearly 20 years ago. A similarly affected sister died, but a lymphoblastoid cell line was available for study. No other affected individuals were known in the Mennonite population. DNA samples were isolated from the proband, her parents, six unaffected siblings, and her deceased sister's cell line. Autozygosity mapping of the two affected individuals identified a single, large block of homozygosity on chromosome 6 (Figure 4, panel A, yellow peak). Analysis of the unaffected siblings excluded this homozygous block, but identified 12 genomic regions greater than 5 Mb in size consistent with linkage in the family (Figure 4, panel A, red peaks, and panel B). None of these regions had large homozygous blocks at 10 K SNP resolution.

Three affected individuals from three separate sibships with *lethal neonatal rigidity and multifocal seizure syndrome* were genotyped originally with 10 K SNP arrays. No significant shared blocks of homozygosity were identified. As the patients were from different yet related Pennsylvania Amish demes, we suspected that the shared homozygous block might be small and thus below the resolution of a 10 K microarray. We subsequently genotyped these patients at 50 K resolution and mapped the disease locus to chromosome 7p22 (Figure 4, panel C).

In 2007, a microcephalic Mennonite baby was evaluated at the CSC and thought to have the same disorder first described by Victor McKusick in 1966 (*microcephaly with chorioretinopathy*, OMIM 251270) [30]. We were able to locate and genotype four affected individuals from McKusick's original study as well as another patient related to our index case. Genotyping at 50 K SNP resolution was required to identify a small 1.8 Mb shared block of homozygosity in the subtelomeric region of chromosome 22q22 (Figure 4, panel D). For both disorders that required higher density arrays, a dearth of SNPs on the 10 K microarray and high recombination rates in these subtelomeric regions complicated mapping.

### Exome Sequencing

Prior to exome analysis, we sequenced between 2 and 45 candidate genes for each condition and found no pathogenic variants. As defined here and throughout the paper, novelty of DNA sequence variants was determined by absence in dbSNP 129 and the 1000 Genomes Project. All putative pathogenic exome variants described below were confirmed by Sanger sequencing in the affected individuals used for genetic mapping. In addition, siblings and parents were also sequenced, when available, to confirm appropriate segregation of the allele within the family. We developed an unlabeled probe melting analysis for each putative pathogenic variant and genotyped population-specific controls for these variants. For each disorder, over 400 population-specific chromosomes were screened, the allele frequencies ranged from 0–1.25%, and no homozygous controls were identified. The exome variant data are summarized in Tables 3 and 4.

**Table 3:** Exome Variant Summary.

	Average Autosomal Variants per Sample			Infantile parkinsonism-dystonia syndrome (n = 1)		Lethal neonatal rigidity and multifocal epilepsy (n = 2)		Microcephaly with chorioretinopathy (n = 1)		Non-syndromic mental retardation (n = 5)		Posterior column ataxia and retinitis pigmentosa (n = 1)		Symptomatic epilepsy and skull dysplasia (n = 3)		Usher syndrome (n = 2)	
				Novel and Homozygous SNPs		SNPs in Mapped Interval		SNPs in Mapped Interval		SNPs in Mapped Interval		SNPs in Mapped Interval		SNPs in Mapped Interval		SNPs in Mapped Interval	
	Total	Novel	Novel and Homozygous	Total	Linked Intervals	Total	Novel	Total	Novel	Total	Novel	Total	Novel	Total	Novel	Total	Novel
**5′-Flanking**	12	1	0														
**5′-UTR**	25	0	0	1										1			
**3′-UTR**	29	1	0														
**Intron**	207	8	1									1				8	
**Missense**	6,936	348	13	9		3		5		5	1	13	1	20	1	39	1
**Nonsense**	46	9	0													1	
**Read-through**	10	1	0					1	1								
**Splice site**	16	1	0	1	1												
**Synonymous**	8,931	220	7	8		4		9		4		9		13		32	
**miRNA**	5	1	0														
**IGR**	56	1	0														
**Other**	62	1	0														
**Indels** *	203	75	*	*	*	2	1										
**Total**	16,540	667	22	19	1	9	1	15	1	9	1	23	1	34	1	80	1

*Novelty for indels measured against dbSNP 129 only, automated indel caller does not determine zygosity.

**Table 4:** Pathogenic Variant Summary.

Disease	Sample Size	Gene	Gene Variant	Protein Variant	Population-specific Allele Frequency
**Infantile parkinsonism-dystonia**	1	*SLC6A3*	IVS9+1G>T	–	0.0% (0/402)
**Lethal neonatal rigidity and seizure syndrome**	3	*BRAT1*	c.638_639insA	–	0.50% (2/402)
**Mental retardation, non-syndromic**	5	*CRADD*	c.382G>C	p.Gly128Arg	1.72% (7/406)
**Microcephaly with chorioretinopathy**	1	*TUBGCP6*	c.5458T>G	p.Ter1820Gly	0.99% (4/404)
**Posterior column ataxia with retinitis pigmentosa**	1	*FLVCR1*	c.361A>G	p.Asn121Asp	1.23% (5/406)
**Symptomatic epilepsy and skull dysplasia**	3	*SNIP1*	c.1097A>G	p.Glu366Gly	1.48% (6/406)
**Usher syndrome**	2	*HARS*	c.1361A>C	p.Tyr454Ser	1.72% (7/406)

#### Infantile Parkinsonism-dystonia syndrome

Exome sequencing was performed on the single living patient. The data were constrained by first tabulating all homozygous, novel variants. Nineteen *novel homozygous* variants were identified: 8 synonymous, 9 missense, 1 5′-UTR, and 1 splice site (Table 3). Of the 10 potentially pathogenic changes, only one localized to a region consistent with linkage in the family, *SLC6A3* IVS9+1G>T. The *SLC6A3* gene encodes the dopamine transporter, a known cause of infantile parkinsonism-dystonia syndrome (OMIM 613135). Direct sequencing revealed both parents were heterozygous for this change, both affected individuals were homozygous, and the unaffected siblings were either heterozygous or homozygous wild-type. This confirmed the linkage block that was identified on chromosome 5 by SNP genotyping. Genotyping of 201 Old Order Mennonite control samples identified no carriers (Table 4).

#### Lethal neonatal rigidity and multifocal seizure syndrome

Exome data from two patients revealed nine variants in the mapped interval, but only one, *BRAT1* c.638_639insA, was novel (Table 3). Genotyping by unlabeled probe melting analysis for 201 Old Order Amish control samples identified 2 carriers (1.0%)(Table 4). Further confirmatory evidence for the pathogenicity of this variant was provided by two unrelated Old Order Amish infants from different demes in Wisconsin and Kentucky who had an indistinguishable clinical phenotype and were homozygous for the *BRAT1* c.638_639insA variant. Their samples were tested for the *BRAT1* variant after local physicians contacted our clinic for clinical guidance.

#### Microcephaly and chorioretinopathy

Exome data from a single affected patient identified 15 sequence variants within the mapped interval. Only one was novel, *TUBGCP6* c.5458T>G (Table 3). An unlabeled probe melting analysis in 202 Old Order Mennonite control samples identified 4 carriers (2.0%)(Table 4). This read-through variant is predicted to incorporate 16 extra amino acids at the C-terminus of TUBGCP6 and/or may accelerate mRNA degradation via non-stop mediated decay.

#### Non-syndromic mental retardation

Exome sequencing of 5 affected individuals identified 9 homozygous sequence variants within the mapped interval. Only a missense variant in *CRADD* (c.382G>C; p.Gly128Arg) was novel (Table 3). PolyPhen-2 predicted this change to be “probably damaging” (score = 0.999). An unlabeled probe melting analysis detected 4 carriers among 203 Old Order Mennonite control individuals (2.0%) (Table 4).

#### Posterior column ataxia and retinitis pigmentosa

Exome data from a single AXPC1 patient revealed 23 coding variants in the mapped interval; 13 were non-synonymous and only one missense variant was novel, *FLVCR1* c.361A>G (p.Asn121Asp) (Table 3). PolyPhen-2 predicts the p.Asn121Asp change to be “probably damaging” (score = 0.997). An unlabeled probe melting analysis for this variant detected 4 heterozygotes out of 203 Old Order Mennonite control chromosomes (2.0%) (Table 4). The pathogenicity of this variant and several others in *FLVCR1* has recently been confirmed independently by others [32].

#### Symptomatic epilepsy and skull dysplasia

Exome data on three affected individuals from two sibships revealed 34 homozygous variants in the mapped region. Of these, only one was novel, *SNIP1* c.1097A>G (p.Glu366Gly) (Table 3). PolyPhen-2 predicts the p.Glu366Gly substitution to be “probably damaging” (score = 0.99). An unlabeled probe melting analysis for this variant identified 5 carriers among 203 Old Order Amish controls (2.5%) (Table 4).

#### Usher syndrome

Exome data from two patients revealed 80 homozygous variants within the mapped interval. Only one variant, *HARS* c.1361A>C (p.Tyr454Ser), was novel (Table 3). Unlabeled probe melting analysis detected 3 carriers among 203 Old Order Amish controls (1.5%) (Table 4). Further evidence for pathogenicity was provided by an Old Order Amish patient who was from an unrelated deme in Ontario, Canada. This Amish settlement arose from a separate migrational event from Europe than the Lancaster County settlement. The patient had an identical phenotype to our patients and was homozygous for the *HARS* c.1361A>C variant.

### In vitro Studies

The *BRAT1* gene encodes BRCA1-associated protein required for ATM activation-1 (BRAT1) [33]. Wild-type human BRAT1 displays prominent nuclear and diffuse cytosolic localization (Figure 1, D) and its C-terminus (amino acids 176–821) interacts with BRCA1 [33]. The c.638_639insA change is a frameshift variant that is predicted to alter the amino acid sequence after Lys^213^ by introducing a stop codon that prematurely truncates the protein at Leu401 (41.5 kDa vs. wild-type 88.1 kDa) (Figure 1, F), abolishes its nuclear localization, and renders the protein unstable in human ARPE-19 cells (Figure 1, E–G). Similar results were obtained with mouse BRAT1 (data not shown). Abundance of the c.638_639insA variant was 87.7±11.9% (n = 3) lower than that of wild-type human BRAT1 for FLAG-fusion proteins overexpressed in human ARPE-19 cells (Figure 1, G). These data suggest that the Amish variant destabilizes the protein and may disrupt an interaction between BRAT1 and BRCA1 that is required for nuclear localization of BRAT1. Knockdown of BRAT1 results in p53-induced apoptosis independent of DNA damage [33]. The destabilization of BRAT1 observed might underlie the catastrophic epilepsy and corticobasal neuronal degeneration observed in affected infants [34], [35], [36], [37], [38].

#### CRADD

CRADD (aka RAIDD) is a caspase-recruitment-domain (CARD) and death domain containing adaptor protein. It links PIDD (p53-induced protein with death domain) and caspase-2 in the formation of the PIDDosome required for caspase-2 activation during apoptosis [39], [40], [41], [42].

**Figure 5:**
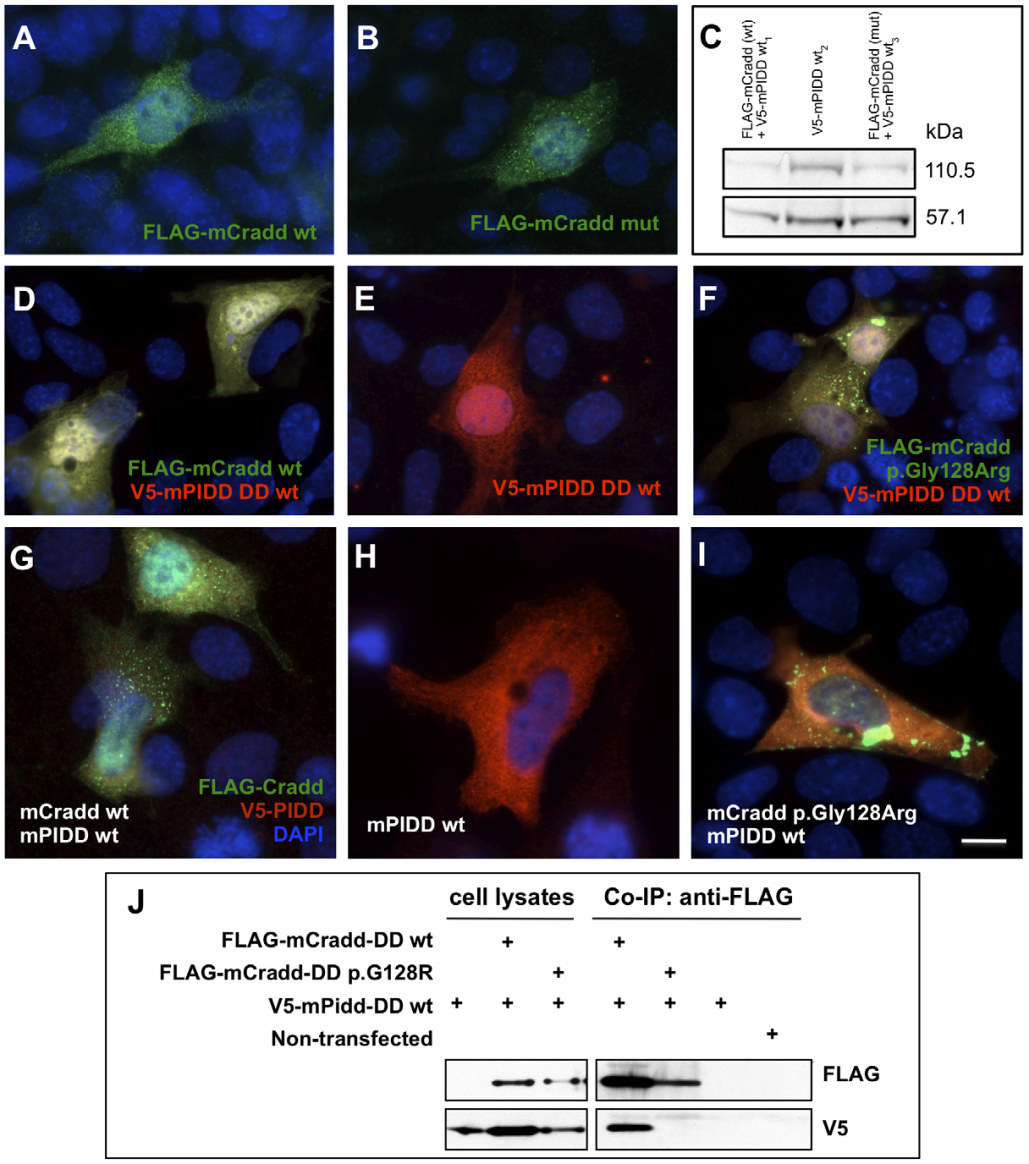
Overexpression of mouse Cradd in mIMCD3 cells. (**A**) Wild-type N-terminal FLAG-Cradd localizes to the cytoplasm and nucleus. (**B**) Mutant N-terminal FLAG-Cradd (p.Gly128Arg) localizes to the cytoplasm and nucleus in a manner that is indistinguishable from the wild-type localization. (**C**) Western blot of lysates from mIMCD3 cells transfected with wt FLAG-Cradd and wt V5-mPIDD, wt V5-mPIDD, or FLAG-Cradd (p.Gly128Arg) and wt V5-mPIDD. Blot was labeled with anti-V5 monoclonal antibody. Note that full-length V5-mPIDD (110.5 kDa) was cleaved into a 57.1 kDa N-terminal fragment, suggesting normal autocatalytic activity. (**D**) Co-transfection of FLAG-Cradd (wt) and wt V5-PIDD death domain (DD) results in uniform colocalization of the fusion proteins throughout the cytoplasm and nucleus (yellow). (**E**) Overexpressed V5-mPIDD DD (wt) localizes to the cytoplasm and the nucleus. (**F**) Co-overexpression of FLAG-Cradd (p.Gly128Arg) with V5-mPIDD DD (wt) results in dense aggregations of FLAG-Cradd in the cytoplasm. (**G**) Co-transfection of FLAG-mCradd (wt) and V5-mPIDD (wt) results in relatively uniform colocalization of the fusion proteins throughout the cytoplasm (yellow); punctate FLAG-mCradd aggregation is also evident (green). FLAG-mCradd (wt) localizes to the nucleus (green), V5-mPIDD does not (red). (**H**) Overexpressed V5-mPIDD (wt) localizes to the cytoplasm as previously reported (Berube et al., 2005). (**I**) Co-overexpression of FLAG-mCradd (p. Gly128Arg) with V5-mPIDD (wt) results in dense aggregations of FLAG-mCradd in the cytoplasm, decreased mCradd∶mPIDD colocalization, and a relative loss of localization of mCradd to the nucleus. Green fluorescence – anti-FLAG M2 antibody (1∶1000); Red fluorescence – anti-V5 antibody (1∶200); Blue fluorescence – DAPI-labeled nuclei (1.5 µg/µl); Western blots – anti-FLAG M2 antibody (1∶1000). (**J**) Co-immunoprecipitation (Co-IP) of V5-tagged mouse Pidd death domain (DD) with FLAG-tagged mouse Cradd DD in mIMCD3 cells. Wild-type FLAG-Cradd DD co-immunoprecipitates wild-type Pidd DD; the FLAG-Cradd DD p.Gly128Arg variant does not. The upper blots were labeled with the anti-FLAG M2 antibody (1∶1000). The lower blots of the same lysates/eluates were labeled with the anti-V5 antibody (1∶5000).

The CRADD c.382G>C mutation alters a highly conserved residue (p.Gly128Arg) within the CRADD death domain. We overexpressed wild-type and mutant (p.Gly128Arg) murine Cradd in mouse inner medullary collecting duct cells (mIMCD3) and found no significant difference in protein localization (Figure 5, A–B) [43]. However, mutant p.Gly128Arg Cradd formed large aggregates when co-overexpressed with wild-type PIDD (Figure 5, I) or the PIDD death domain (Figure 5, F). We found that overexpressed PIDD localized to cytosol (Figure 5, H) as described previously [43] and was autocatalytically cleaved as expected (Figure 5, C) [42]. These data suggest that the p.Gly128Arg mutation alters one of the interaction surfaces of the CRADD death domain to decrease affinity for the PIDD death domain and increase homotypic binding to the CRADD death domain [44]. This is further supported by co-immunoprecipitation assays demonstrating that wild-type Cradd DD co-immunoprecipitates Pidd DD, but Cradd DD p.Gly128Arg does not (Figure 5, J). In rat PC-12 cells and cultured sympathetic neurons, similar perinuclear Cradd aggregations are associated with caspase-2 activation and initiation of apoptosis [45]. In humans, alteration of caspase-2 initiated apoptosis (resulting from disruption of the PIDDosome) during nerve growth factor mediated proliferation of synaptic contacts may lead to inappropriate synaptic pruning that results in cognitive impairment.

#### SNIP1

Wild-type SNIP1 (Smad nuclear interacting protein 1) contains an N-terminal nuclear localization signal [46], bridges c-Myc activity with CBP/p300 activity during development, and competitively inhibits TGF-β and NF-κB signaling [47], [48]. SNIP1 Glu^366^ is highly conserved and the p.Glu366Gly mutation is in the C-terminus where SNIP1 interacts with c-Myc [48], Smad1 and Smad2 [46], [49].

Reverse transcriptase-PCR suggests that the *Snip1* c.1058A>G (corresponding to human *SNIP1* c.1097A>G) transcript is expressed at levels comparable to wild-type (Figure 3, F). We transiently overexpressed wild-type and mutant Snip1 in mIMCD3 cells. Wild-type protein localized to the nucleus with a punctate appearance, consistent with its involvement in transcriptional complexes [46], [48] (Figure 3, D). However, Snip1 p.Glu353Gly (corresponding to human p.Glu366Gly) had a more aggregated appearance (Figure 3, E) and Western blotting proved it unstable (Figure 3, F); its band density was 84.9±9.6% SD lower than wild-type (n = 4 independent transfections each).

Decreased abundance of SNIP1 likely results in decreased c-Myc activity and increased TGF-β and NF-κB signaling. Disruption of c-Myc or CBP/p300 signaling in mice can result in abnormal development of the brain, skull, craniofacial bones, and distal limbs [50], [51], [52], [53], [54]. Inappropriate increases in TGF-β/Smad2 signaling and in NF-κB signaling in the mouse brain have been shown to independently initiate epileptogenesis [55], [56].

#### HARS

**Figure 6:**
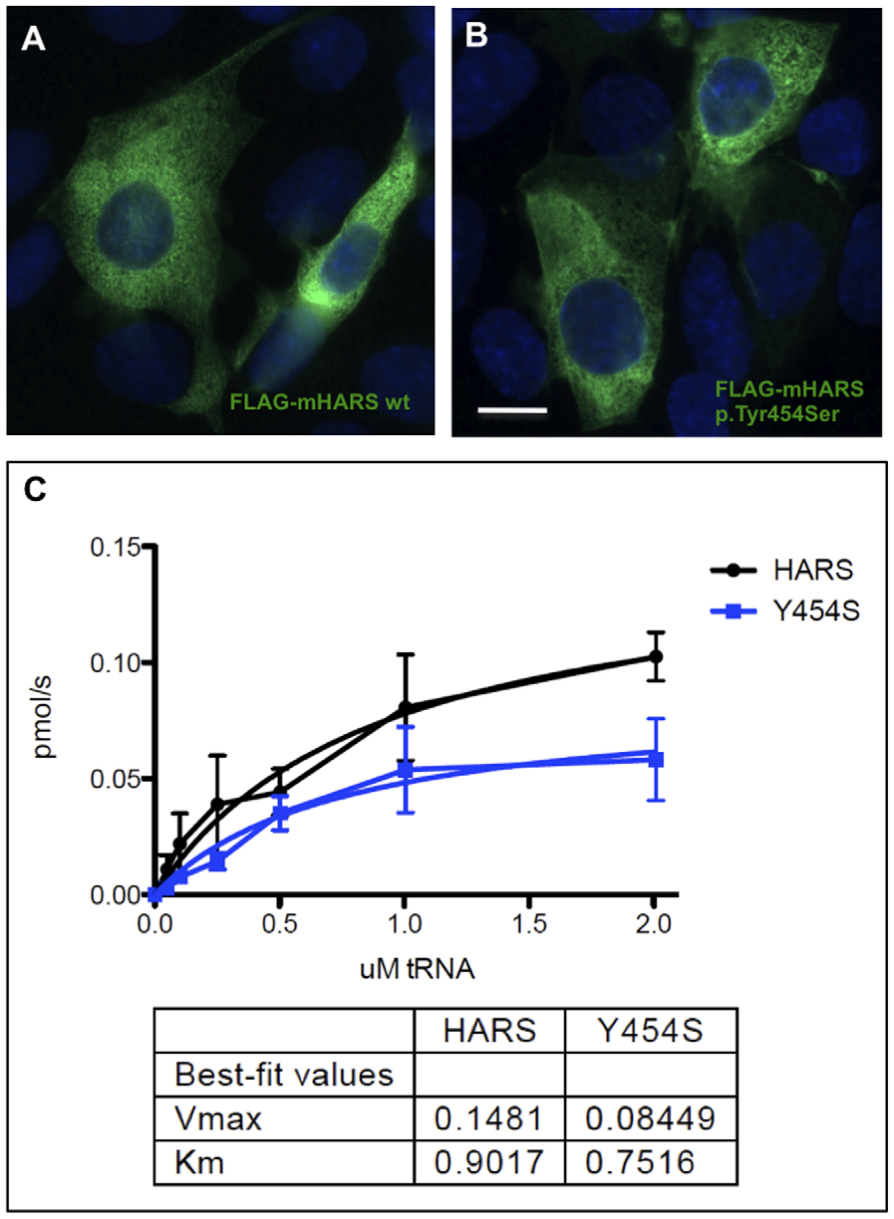
Overexpression of mouse HARS in mIMCD3 cells. (**A**) Wild-type N-terminal FLAG-HARS localizes to the cytoplasm. (**B**) Mutant N-terminal FLAG-HARS (p.Tyr454Ser) localizes to the cytoplasm in a manner that is indistinguishable from the wild-type localization shown in **A**. Transfected and non-transfected cells were labeled with anti-FLAG M2 monoclonal antibody and AlexaFluor 488-conjugated anti-mouse IgG_1_ (green fluorescence). (**C**) Reaction velocity vs. human tRNA^His^ concentration for histidine aminoacylation of tRNA^His^ by wild-type murine HARS (HARS) and p.Tyr454Ser (Y454S) HARS. Scale bar = 10 µm in **A** and **B**.

Histidyl-tRNA synthetase (HARS/HisRS) is a homodimeric class IIa aminoacyl tRNA synthetase that charges tRNA with the amino acid histidine [57]. The p.Tyr454Ser change rests in the interface between the catalytic domain and anticodon binding domain suggesting that it may alter anticodon recognition and/or catalytic activity. Overexpression of wild-type and mutant (p.Tyr454Ser) murine HARS in mIMCD3 cells did not reveal any significant differences in localization (Figure 6, A–B). In Chinese hamster ovary (CHO) cell lysates, both wild-type and mutant murine protein dimerized properly with endogenous HARS and were expressed at qualitatively similar levels (data not shown). Our data suggest that the p.Tyr454Ser change reduces the maximal forward reaction velocity (Vmax) of the enzyme for aminoacylation of human tRNAHis with histidine nearly two-fold (Figure 6, C).

## Discussion

Next generation sequencing technologies promise to expedite disease gene discovery and allowed us to identify known and novel pathogenic variants in our patients. Although costly, exome sequencing is practical as it interrogates the 1.5% of the genome that contains approximately 95% of pathogenic variants [12], [13], [14], [15], [16], [17], [18], [19], [20], [21], [22], [23], [24], [25], [26], [27], [28]. To assess the utility of exome sequencing in an active clinical setting, we selected 15 patient samples representing 7 different genetic conditions. Each disorder had previously been mapped to a chromosomal locus and candidate gene sequencing failed to identify the pathogenic variant. For six disorders, an average autozygous block of 4.4 Mb (0.13% of the human genome) contained only one novel homozygous variant and rendered disease gene identification straightforward. Even in the case of infantile parkinsonism-dystonia syndrome, for which autozygosity and linkage mapping were only partially informative, a manageable list of 19 candidate variants was assembled simply by assuming mutation homogeneity. Prior mapping data and thorough knowledge of the patient implicated a single variant from this list (*SLC6A3* IVS9+1G>T).

For four of the conditions, more than one affected individual was available for exome sequencing. Even in the absence of mapping data, the identification of the putative pathogenic variant would still have been unambiguous. When we examined the shared, novel homozygous variants in affected individuals, we found one, and only one, that was not homozygous in any other (unaffected) individuals in the study. Thus, the assumption of mutation homogeneity obviates the need for SNP genotyping and mapping; we reach the same conclusion by exome sequencing of multiple affected individuals without the added time and expense of SNP genotyping.

The number of novel homozygous variants in each individual was surprisingly small. Average inbreeding coefficients of 4% and 2.5% in the Lancaster Amish and Mennonite populations, respectively, suggested that a small but significant fraction of variation will be homozygous. On average, we found only 21 novel homozygous variants per sample across the exome. Of these variants, only 12 were predicted to be potentially pathogenic (missense, nonsense, splice site). This represents 3.7% of all novel variants per exome. For the two disorders where a singleton was sequenced, we identified only 6 potentially pathogenic novel variants which were homozygous in the patient but in no other samples. Since our total sample size was small (15 individuals), we expect that future studies which leverage accumulated exome data will allow us to sequence single individuals to identify rare, uniquely homozygous pathogenic variants. In the outbred population, a strategy that scans for homozygosity or compound heterozygosity for novel variants in the same gene should yield equally manageable candidate gene lists.

Among the fifteen individuals studied, we found 4200 different novel autosomal sequence variants, roughly 62% of which have pathogenic potential. We infer that 3.6% of these variants were non-pathogenic changes as they were homozygous in one or more unaffected individuals. As more Amish and Mennonite exomes are analyzed within a clear clinical context, our ability to determine pathogenicity will improve. The exome data also provided a broader view of the genetic disease burden within these populations. We have catalogued 94 known pathogenic sequence variants within the Plain populations that should be detectable by exome sequencing. Of these, 11 were represented in at least one individual. On average, each individual harbored 1.4 known Plain pathogenic alleles (range, 0–4). We also compared our exome results against the Human Gene Mutation Database (HGMD) [58]. Carrier status for 113 HGMD mutations, that cause phenotypes not yet encountered in the Plain populations, was detected in our patients. These data permit us to generate a more comprehensive molecular differential diagnosis list when faced with a new clinical phenotype. It is notable that several HGMD-DM mutations were homozygous in one or more patients, casting some doubt on the pathogenicity of these variants. Similar results have been reported elsewhere and highlight the need for better curation of mutation databases [59].

Critics will argue that we have failed to exclude all possible variants due to incomplete coverage. Our sequencing metrics show excellent, albeit incomplete, exome coverage. On average, 91.8% and 86.6% of the targeted exome was sequenced to a depth of 10× and 20×, respectively. While this is a potential hazard, our study design minimized this risk. Prior mapping analyses narrowed the focus to a vanishingly small 0.13% of the genome. Within these mapped regions, we discovered only one novel homozygous variant. This is significantly better coverage and stronger evidence than we and others have demonstrated for disease gene identification prior to the advent of exome sequencing. Additionally, our SNP filtering strategies might be questioned; dbSNP is polluted with many pathogenic variants and their numbers continue to grow as data accrues. Our current conservative strategy used dbSNP 129 and the 1000 Genomes Project to filter exome variants. The risk to our analyses is relatively small since we study very rare and highly penetrant alleles. Nonetheless, in our population and elsewhere, local population-specific variant databases will prove most useful for inferring pathogenicity.

Pathogenicity is difficult to prove, but for four conditions we provide ample functional data to demonstrate abrogation of protein function. In vitro studies of protein localization and function in mammalian cells provide further confirmation that the homozygous variants identified were indeed pathogenic. The predicted consequence of the *BRAT1* c.638_639insA frameshift variant is a truncated protein at amino acid position 401. Overexpression of this truncated protein abolished nuclear localization and demonstrated protein instability (87.7% decrease relative to wild-type). An alternative disease mechanism, nonsense-mediated mRNA decay, has not been investigated. Others have shown that knockdown of BRAT1 results in p53-induced apoptosis [33]. This is consistent with the neurodegeneration observed in patients with the *BRAT1* variant. When transfected into mouse IMCD3 cells, the mouse counterpart of the *CRADD* c.382G>C variant disrupts interaction with mouse Pidd (it's normal binding partner) and forms dense aggregates when co-expressed with wild-type mouse Pidd. This is in contrast to a pattern of uniform colocalization of wild-type Cradd and Pidd throughout the cytoplasm and nucleus. Mouse Snip1 protein, when transiently overexpressed in IMCD3 cells, localizes to the nucleus in a punctate pattern consistent with transcriptional complexes, while mutant mouse Snip1 (p.Glu353Gly, corresponding to human p.Glu366Gly) localizes to the nucleus, but with a more aggregated distribution. Western blotting proved this structure unstable. For the *HARS* c.1361A>C variant, we demonstrate that reaction velocity (Vmax) for aminoacylation of human tRNAHis with histidine is reduced nearly two-fold. While these studies cannot prove pathogenicity beyond a shadow of doubt, the totality of evidence is compelling and strongly suggests that we have identified the disease-causing alleles. The association between *FLVCR1* and *SLC6A3* variants and disease has previously been established.

We provide no further evidence for pathogenicity of the *TUBGCP6* c.5458T>G variant. However, the primary microcephalies result from disruption of the centrosomal complex during mitosis, reducing the neural progenitor pool during development (39–41). Centrosomal proteins such as CDK5RAP2 and CENPJ/CPAP interact with the γ-tubulin ring complex (γ-TuRC) to regulate microtubule nucleation (42–44). TubGCP6 (GCP6) is a component of the human γ-TuRC (45), where it is required for CDK5RAP2 to activate microtubule nucleation (43). We predict that defects in *TUBGCP6*, *CDK5RAP2* and *CENPJ* cause primary microcephaly by similar mechanisms.

All seven variants described in this paper interfere with neurological development. Functional studies of BRAT1 and SNIP1 will expand our understanding of epilepsy and also deepen our knowledge of DNA damage repair and transcriptional regulation in cortical development and neuronal survival. Selective degeneration of photoreceptors and dorsal column afferents caused by *FLVCR1* mutations suggest an unusual vulnerability of these cells to deranged heme transport, or may reveal an altogether different function of the FLVCR1 protein. Despite its ubiquitous expression, a defect in aminoacylation by HARS selectively damages elements of afferent sensory systems and, by unknown mechanisms, predisposes to episodic psychosis and sudden death. Abnormalities of neuroblast proliferation and migration caused by *TUBGCP6* mutations fit nicely with our existing knowledge about centrosomal complexes, microtubular arrays, and cortical growth, but also introduce new questions about the diverse brain morphologies linked to various specific tubulin-associated proteins and the role of these proteins in early eye development. Finally, further studies on the connection between CRADD and general intelligence will certainly change our understanding of the microanatomical and molecular bases of cognition. Small focused studies as described herein will be a steady engine of progress for understanding the specific connections between genes and the human brain.

Ultimately, genomics can only shape medical practice within the context of regional particulars and clinical facts. Our local, patient- and family-based approach to gene discovery stands in stark contrast to the prevailing model of genomic research, where the people who produce genotype data are frequently separated from those who collect and analyze clinical facts, and both struggle to translate genetic knowledge into primary care. Although we are focused on specific regional populations (1, 3), these studies reveal concepts of broad biological and economic relevance (8, 11). The discovery of rare, highly penetrant alleles among small social groups may prove more useful than large genome-wide association studies for revealing the basic genetic foundations of complex disease, particularly when these alleles can be viewed against a background of population-specific genetic variation. Even at current prices, microarray analyses to detect copy number abnormalities coupled with exome sequencing are an order of magnitude cheaper than the standard workup for a complex patient at a tertiary medical center. Thoughtful and appropriately scaled application of these genetic technologies to other regional populations should yield similar economic and clinical benefits in the years ahead.

## Materials and Methods

The study was approved by Institutional Review Boards at Lancaster General Hospital and the Broad Institute. All parents consented to participation. Patients were from Old Order Amish or Mennonite populations, received medical care at the Clinic for Special Children (CSC), and presented with a distinctive recessive clinical phenotype (Table 1).

### Genetic Mapping

Genetic mapping of the seven disorders presented in this study was performed as previously described [5], [6], [7], [8], [9], [10], [11] with the exception that two disorders required higher density arrays (Affymetrix 50 K) to establish chromosomal location. Single-nucleotide polymorphism (SNP) genotyping was performed with the GeneChip Mapping 10 K and 50 K Assay Kits (Affymetrix, Santa Clara, CA, USA) as previously described. Data were analyzed in Microsoft Excel spreadsheets (Microsoft Corporation, Redmond, WA, USA) that were custom formatted at the Clinic for Special Children. SNP positions came from Affymetrix genome annotation files and genotype data came from the Affymetrix GeneChip Human Mapping 10 K Xba 142 and 50 K Xba Arrays. Data analyses were designed for rapid identification of genomic regions that were identically homozygous between all affected individuals. These analyses assumed mutation and locus homogeneity. Two-point lod scores were calculated for each genotyped SNP using an approach similar to Broman and Weber [60]. Cumulative two-point lod scores for blocks of homozygous SNPs were considered the location score for that region, providing a relative measure that a specific homozygous block harbored the disease gene. Genotype data from 100 healthy population-specific (Amish or Mennonite) females were used for estimation of SNP allele frequencies.

### Exome Sequencing

Exome sequencing was performed using the Agilent SureSelect All Exon Kit (v.1, 38 Mb) as previously described [*61*]. A solution hybrid selection methodology was used to isolate exomic DNA, which was subjected to sequencing on the Illumina GA-II platform. Briefly, DNA oligonucleotides, corresponding to 120 bp of target sequence flanked by 15 bp of universal primer sequence, were synthesized in parallel on an Agilent microarray, then cleaved from the array. The oligonucleotides were PCR amplified, then transcribed in vitro in the presence of biotinylated UTP to generate single-stranded RNA “bait.” Genomic DNA was sheared, ligated to Illumina sequencing adapters, and selected for lengths between 200–350 bp. This “pond” of DNA was hybridized with an excess of bait in solution. The “catch” was pulled down by magnetic beads coated with streptavidin, then eluted, and sequenced on the Illumina GA-II.

Massively parallel sequencing data were processed by the Sequencing Platform at the Broad Institute using two consecutive pipelines. The first pipeline, called “Picard”, utilized the reads and qualities produced by the Illumina software for all lanes and libraries and produced a single BAM file (http://samtools.sourceforge.net/SAM1.pdf) representing a sample. The final BAM file stored all reads with well-calibrated qualities and alignments to the genome. The second pipeline, called Genome Sequencing Analysis, then performed post-processing and analysis of the data including SNP identification, small insertion and deletion identification, local realignment of insertion or deletion containing reads, gene annotation and filtering with common polymorphisms (1000 Genomes, dbSNP build 129). The details of our sequencing data processing have been described elsewhere [62]. Inter-exome analyses were performed by importing variant call data into a FileMaker Pro database (FileMaker, Inc., Santa Clara, CA) and querying mapped intervals for novel variants shared among affected individuals.

### Candidate Disease Gene Cloning

Total RNA extracted with Trizol (Invitrogen, Carlsbad, CA) from wild-type mouse tissues, cultured mouse IMCD3 cells, or human ARPE19 cells was reverse transcribed with SuperScript II reverse transcriptase (Invitrogen). Gene specific primers were used to amplify full-length cDNA (coding sequence, CDS) for the candidate disease genes by the polymerase chain reaction (PCR). Mouse PIDD was amplified from pcDNA3.1(−)/mPIDD (a gift from S. Benchimol, York University, Toronto, Ontario, Canada). Constructs were ligated into D-TOPO Gateway entry vectors (Invitrogen) for cloning, and site-directed mutagenesis (QuikChange II, Agilent, Santa Clara, CA) was used to introduce the mutations discovered by exome sequencing. Wild-type and mutant constructs (confirmed by Sanger sequencing of the full-length inserts) were subcloned into pCAG/FLAG/RFC/A and/or pCAG/V5/RFC/A (gift of Q. Zhang and E.A. Pierce, University of Pennsylvania, Philadelphia, PA) for expression of N-terminal FLAG- and/or V5-fusion proteins in mammalian cells.

### Immunohistochemistry

For protein localization studies, mIMCD3 cells grown to 80–90% confluence on glass coverslips in six-well plates were transfected with FLAG and/or V5-fusion protein expression vector plasmid DNA using Lipofectamine 2000 (Invitrogen). Cells were grown for 40–48 h post-transfection and then fixed for 10 min in 4% paraformaldehyde in phosphate buffered saline (PBS), permeabilized for 10 min with 0.5% Triton X-100 in PBS, blocked for 10 min with 1% bovine serum albumin in PBS with 0.2% Triton X-100, and incubated with primary antibodies for 1 h at room temperature (RT). Following a wash in PBS, cells were incubated in secondary antibodies for 1 h at RT. Nuclei were counterstained with DAPI (4′,6-diamidino-2-phenylindole – 1.5 µg/µl; Santa Cruz Biotechnology, Santa Cruz, CA) and coverslips were mounted with Fluoromount G (Electron Microscopy Sciences, Ft. Washington, PA). Fluorescent images were captured with a Leica DM RB microscope. Primary antibodies – monoclonal anti-FLAG M2 IgG_1_ (1∶1000; Sigma, St. Louis, MO) and/or monoclonal anti-V5 IgG_2a_ (1∶200; Invitrogen). Secondary antibodies – DyLight 488-conjugated goat anti-mouse IgG (1∶400; Jackson ImmunoResearch, West Grove, PA), AlexaFluor 488-conjugated goat anti-mouse IgG_1_ (1∶400), and/or AlexaFluor 594-conjugated goat anti-mouse IgG_2a_ (1∶400; Invitrogen).

### Western Blotting, Co-immunoprecipitation, and RT-PCR

Western blotting was used to assess relative protein overexpression levels. Briefly, 100% confluent mIMCD3 cells in six-well or 10-cm plates transfected as described above were lysed in either 4× LDS sample buffer (Invitrogen) or in lysis buffer (CelLyticM, Sigma) containing protease inhibitor and phosphatase inhibitor cocktails (Roche Applied Science, Indianapolis, IN). Lysates were sonicated briefly and cleared by centrifugation at 14000 rpm for 10–15 min. Following SDS-PAGE, proteins were blotted to nitrocellulose, and the membranes were blocked for 1 h at RT with 5% nonfat dry milk in Tris buffered saline (TBS) with 0.1% Tween-20. Membranes were then incubated with primary antibody (anti-FLAG M2 – 1∶1000 or anti-V5 – 1∶5000) overnight at 4°C, washed in TBS with 0.1% Tween-20, and then incubated with secondary antibodies for 1 h at RT. Secondary antibodies – horseradish peroxidase (HRP)- conjugated goat anti-mouse IgG (1∶1500) and HRP-conjugated anti-biotin (1∶1000). Proteins were detected with enhanced chemiluminescence using LumiGLO (Cell Signaling Technology, Danvers, MA) and chemiluminescence signals were imaged on BioMax Light film (Kodak, Rochester, NY). Band densities were measured with ImageJ (http://rsbweb.nih.gov/ij/) following calibration with a NIST calibrated step tablet (Kodak EK1523406).

Co-immunoprecipitation was carried out using the FLAG-M2 affinity resin according to the manufacturer's protocols (Sigma CELLMM2/F2426) with the following exception: cleared lysates were incubated at 37°C for 1 h prior to overnight incubation on the resin.

Reverse transcriptase-PCR was used to assess relative transcript levels for genes overexpressed in cultured mIMCD3 cells. Cells were transfected on 10-cm plates and cultured as described above. Total RNA was harvested, incubated with DNase (1 U/µg RNA) at 37°C for 30 min, and reverse transcribed as described above. A FLAG-tag specific forward primer and gene specific reverse primers were used to determine the transcript levels for the overexpressed gene relative to endogenous GAPDH or β-actin expression using 20–24 cycles of PCR. Total transcript levels (endogenous+overexpressed) for the gene of interest were determined with gene specific primer pairs for short (300–500 bp) amplicons relative to GAPDH expression.

### Aminoacylation assays

Murine HARS and the variant p.Tyr454Ser HARS were expressed in Chinese hamster ovary (CHO) cells as FLAG-tagged enzymes and affinity purified. Enzymes were stored at −20°C in 34 mM Tris HCl, pH 7.4, 100 mM NaCl, 50% glycerol at a concentration of 10 µM and diluted to 1 µM in cold reaction buffer (50 mM HEPES pH 7.5, 100 mM KCl, 10 mM MgCl2 and 1 mM DTT) just before use. Human tRNAHis, which has an identical sequence to murine tRNAHis, was prepared from human placenta as described, with minor modifications [63]. Isolated placental RNA was stored at −20°C in 10 mM Na Cacodylate (pH 6.0). This preparation yields approximately 25 µg RNA/g tissue, of which 1.4% (22.4 pmol/A260 unit) can be aminoacylated with histidine by murine HARS.

### Kinetics assay

Enzyme assays were performed under steady state conditions for ATP and histidine, with the concentration of tRNA varied from approximately 0.2 to 5× KM, as previously described [64], [65]. Briefly, ATP (2.5 mM), [3H] histidine mixed with histidine (25 µM, 19% radiolabeled), pyrophosphatase (10 ng/µl) and buffer (50 mM HEPES pH 7.5, 100 mM KCl, 10 mM MgCl2 and 1 mM DTT) were mixed with placental tRNA (35.9 to 402 µM, of which 1.4% could be histidylated). The reaction was initiated by addition of enzyme (100 nM), and quenched by spotting on filter paper pre-soaked with 10% trichloroacetic acid. After washing away unbound radiolabeled histidine, the histidylated tRNA was quantified by scintillation counting. The time course reactions were analyzed using KinTek Explorer software, yielding a velocity versus substrate curve from which kinetic parameters were determined.
